# Cardiovascular magnetic resonance with late gadolinium enhancement improves mortality prediction beyond echocardiography: a comparative effectiveness study

**DOI:** 10.1186/1532-429X-14-S1-O34

**Published:** 2012-02-01

**Authors:** Erik B Schelbert, Diego Moguillansky, Timothy C Wong

**Affiliations:** 1 University of Pittsburgh, Pittsburgh, PA, USA

## Summary

Adding information from CMR with LGE to ejection fraction measured by echocardiography significantly improves the prediction for all cause mortality and better classifies individuals at risk.

## Background

We tested the hypotheses that 1) ejection fraction (EF) assessed by cardiovascular magnetic resonance (CMR) is a stronger predictor of all cause mortality than EF assessed by echocardiography; and 2) CMR with late gadolinium enhancement (LGE) improves risk stratification over echocardiography EF in Cox regression models as determined by net reclassification improvement (NRI). Echocardiography with EF measurement remains the initial noninvasive imaging modality for evaluation of patients, and it is used to individualize medical, surgical, and device-based therapy. Yet, if CMR with LGE improves risk stratification compared to echocardiography, then CMR would demonstrate a potential to optimize care through improved patient selection for interventions and more efficient allocation of healthcare resources.

## Methods

We prospectively enrolled 1076 consecutive patients referred for clinical CMR and identified those (n=444) with contemporaneous (≤ 3 weeks apart) echocardiography. Those with confounders such as tako-tsubo cardiomyopathy or with echocardiography >6 days apart in the setting of acute myocardial infarction were excluded (n=7). Baseline characteristics were acquired through interview and medical record documentation. EF and LGE were obtained from the final report and correlated in linear regression models. Vital status was ascertained from the Social Security Death Index and medical record review. Cox regression models quantified the hazards associated with EF decrements, and the NRI identified whether LGE added prognostic value to covariates contained in Cox models.

## Results

Echocardiography EF and CMR EF were only modestly correlated (R2=0.66, p<0.001) with minimal bias on Bland-Altman analysis. There were 30 deaths (6.8%) over a 2 year follow-up period (median 9 months) CMR EF was more strongly associated with mortality than echocardiography EF (χ2: 21 vs. 17). LGE was strongly related to mortality when added to a Cox model with echo EF (HR 6.15; 95%CI 1.84-20.5) even when adjusted for age, gender, and diabetes (HR 4.05; 95%CI 1.17-14.0). LGE improved the net reclassification of patients at risk for death: NRI=0.75; 95% CI 0.44-1.00 for LGE added to Cox model with echocardiography EF; and NRI=0.49; 95%CI 0.08-0.84 for LGE added to Cox model with echocardiography EF, age, gender, diabetes.

## Conclusions

EF measured by CMR provides superior risk stratification and better predicts subsequent mortality compared to EF by echocardiography. CMR with LGE improves the classification of individuals at risk for death even if contemporaneous echocardiography derived ejection fraction is available. These data may have implications for efforts to optimize processes of care, assess comparative effectiveness, and allocate limited health care resources.

## Funding

Dr Schelbert is supported by a Pittsburgh Foundation grant and an American Heart Association Scientist Development grant (09SDG2180083) including a T. Franklin Williams Scholarship Award; funding provided by: Atlantic Philanthropies, Inc, the John A. Hartford Foundation, the Association of Specialty Professors, and the American Heart Association. Dr. Wong is supported by a grant K12 HS19461-01 from the Agency for Healthcare Research and Quality.

